# Wnt/β-Catenin Signaling Induces the Aging of Mesenchymal Stem Cells through the DNA Damage Response and the p53/p21 Pathway

**DOI:** 10.1371/journal.pone.0021397

**Published:** 2011-06-21

**Authors:** Da-yong Zhang, Hai-jie Wang, Yu-zhen Tan

**Affiliations:** Department of Anatomy and Histology and Embryology, Shanghai Medical College of Fudan University, Shanghai, People's Republic of China; Baylor College of Medicine, United States of America

## Abstract

Recent studies have demonstrated the importance of cellular extrinsic factors in the aging of adult stem cells. However, the effects of an aged cell–extrinsic environment on mesenchymal stem cell (MSC) aging and the factors involved remain unclear. In the current study, we examine the effects of old rat serum (ORS) on the aging of MSCs, and explore the effects and mechanisms of Wnt/β-catenin signaling on MSC aging induced by ORS treatment. Senescence-associated changes in the cells are examined with SA-β-galactosidase staining and ROS staining. The proliferation ability is detected by MTT assay. The surviving and apoptotic cells are determined using AO/EB staining. The results suggest that ORS promotes MSC senescence and reduces the proliferation and survival of cells. The immunofluorescence staining shows that the expression of β-catenin increases in MSCs of old rats. To identify the effects of Wnt/β-catenin signaling on MSC aging induced with ORS, the expression of β-catenin, GSK-3β, and c-myc are detected. The results show that the Wnt/β-catenin signaling in the cells is activated after ORS treatment. Then we examine the aging, proliferation, and survival of MSCs after modulating Wnt/β-catenin signaling. The results indicate that the senescence and dysfunction of MSCs in the medium containing ORS is reversed by the Wnt/β-catenin signaling inhibitor DKK1 or by β-catenin siRNA. Moreover, the expression of γ-H2A.X, a molecular marker of DNA damage response, p16^INK4a^, p53, and p21 is increased in senescent MSCs induced with ORS, and is also reversed by DKK1 or by β-catenin siRNA. In summary, our study indicates the Wnt/β-catenin signaling may play a critical role in MSC aging induced by the serum of aged animals and suggests that the DNA damage response and p53/p21 pathway may be the main mediators of MSC aging induced by excessive activation of Wnt/β-catenin signaling.

## Introduction

Stem cells are important for maintaining and repairing adult organs. Recent data have demonstrated that the stem cells of older individuals show senescence and their function gradually decline with increasing age [1]. Mesenchymal stem cells (MSCs) are characterized by their ability to self-renew and to differentiate into multiple cell lineages [2], [3], and have been widely used in clinical cell transplantation therapy [4]. However, the aging of MSCs affects their clinical application [5], [6]. Recent studies have shown that MSC function declines in older individuals and that MSC dysfunction influences the effects of autologous MSC transplantation in older individuals [7], [8]. Moreover, when xenogenic MSCs are transplanted in older individuals, MSC function is also limited in the older recipients because of the effects of the aged cell–extrinsic environment [9]. Increasing studies have shown that an aged cell–extrinsic environment plays an important role in the aging of adult stem cells [10]–[12]. However, the effects of an aged environment on MSC function, especially on their ability to proliferate and survive, remain unclear. Therefore, research on the effects of an aged cell–extrinsic environment on the senescence and function of MSCs has important clinical significance.

A number of studies have demonstrated that serum is an important factor in cell senescence [13], [14]. As a systemic milieu [15], serum has an important influence on stem cell function [16]. Recent studies have suggested that old mouse serum induces the aging or dysfunction of satellite cells, embryonic stem cells, and hemopoietic stem cells [11], [13], [14]. However, the critical factors that promote stem cell aging in the serum of older individuals are still unclear. Brack et al. [17] investigated the effects of aged cell–extrinsic environment on satellite cell senescence or dysfunction in a parabiosis model, in which the animals develop a common circulatory system, allowing blood to move between the young and old mice. When young mice are parabiotically fused with older mice, the Wnt activity of satellite cells in young mice increases, which suggests that the Wnt/β-catenin signaling of satellite cells in young mice is activated by the serum of old mice. However, more experimental evidence is necessary to identify the relationship between Wnt/β-catenin signaling and the stem cell aging induced by an aged systemic milieu.

Wnt/β-catenin signaling is activated by the binding of Wnt ligands to the frizzled family of receptors. In the absence of Wnt ligands, β-catenin is phosphorylated by glycogen synthase kinase-3β (GSK-3β) and then degraded by the ubiquitin-proteasome system. When Wnt ligands bind to frizzled receptors, GSK-3β activity is inhibited, and unphosphorylated β-catenin accumulates in the cytoplasm and translocates into the nucleus, where it promotes the transcription of a variety of the target genes (such as c-myc) [18]. In adult mammals, Wnt/β-catenin signaling is crucial for regulating cell proliferation, cell fate determination, apoptosis, and axis polarity induction [19]. Some recent studies have shown that Wnt/β-catenin signaling is involved in cellular senescence. Liu et al. [20] investigated the effects of Wnt signaling on stem cell aging in klotho knockout mice, and demonstrated that klotho mutant mice have elevated Wnt activity, which accelerates the senescence of stem cells in hair follicles, bones, and intestinal crypts. Other studies have shown that constitutive activated Wnt/β-catenin signaling leads to the rapid exhaustion of hematopoietic stem cells [21], [22], and the senescence or dysfunction of fibroblasts [23], thymocytes [24], endothelial cells [25], and human mammary artery cells [26]. These data have revealed the new biological role of Wnt/β-catenin signaling on cellular aging. However, whether the Wnt/β-catenin signaling plays an important role in MSC aging remains unclear. In the present study, we investigate the effects of the Wnt/β-catenin signaling on MSC aging.

The mechanisms of cell senescence induced by Wnt/β-catenin signaling are still poorly understood. Xu et al. [24] reported that β-catenin overexpression induces γ-H2A.X expression in thymocytes, which suggests that activated Wnt/β-catenin signaling can induce the DNA damage response (DDR). DDR induces p16^INK4a^ expression [27], [28]. The p16^INK4a^ gene is an aging-induced gene that directly induces cellular aging [29], [30]. Other studies have shown that activated Wnt/β-catenin signaling causes p53 accumulation [23], [25], whereas p21, a target gene of p53 protein, directly induces cellular aging [31]. Therefore, we detected the expression of γ-H2A.X, p16^INK4a^, p53, and p21 in MSCs, and explored whether activated Wnt/β-catenin signaling induces aging in MSCs through DDR and the p53/p21 pathway.

In the current study, the effects of old rat serum (ORS) on aging, proliferation, and survival of MSCs are investigated. To determine the effects of ORS on Wnt/β-catenin signaling of MSCs, the expressionof β-catenin, GSK-3β, and c-myc are examined. In addition, to evaluate the role of Wnt/β-catenin signaling on MSCs aging, we investigated the aging, proliferation, and survival of MSCs after modulating Wnt/β-catenin signaling. Finally, to determine the mechanisms of Wnt/β-catenin signaling in MSCs aging, the expression of γ-H2A.X, p16^INK4a^, p53, and p21 are examined.

## Materials and Methods

### Isolation and culture of MSCs

Sprague–Dawley (SD) rats were obtained from the Medical Institute Animal Center of Fudan University (Permit number SYXK (Shanghai) 2009-0019), China. The investigation was permitted by the Law of the People's Republic of China on the Protection of Wildlife, and the protocol was approved by the Institutional Animal Care Committee from Fudan University, China. Bone marrow was harvested from SD rats aged 12–14 weeks. The femurs and tibias were removed from the SD rats and bone marrow was flushed out of the bones using 10 mL PBS with 100 U/mL heparin in a syringe. The cells were centrifuged at 1000 rpm for 8 min. The cell pellet was resuspended in 10 mL Dulbecco's modified Eagle's medium (DMEM; Gibco, USA) supplemented with 15% fetal bovine serum (FBS, Gibco, USA) and plated in a 25 cm^2^ plastic flask (Corning, USA) to allow the MSCs to adhere. After 3 days, the medium was changed and the non-adherent cells were discarded. The medium was completely replaced every three days. Approximately 7–10 days after seeding, the cells became nearly confluent. The adherent cells were released from the dishes with 0.25% trypsin (Gibco, USA) and seeded into new fresh culture flasks. All the experiments described below were performed using MSCs from the third to the fifth passage.

### Isolation of serum

Whole blood was collected from anesthetized young (3–4 weeks old) or old (64–68 weeks old) SD rats via the abdominal aorta. Blood was clotted at 37°C for 4 h. Serum was isolated by centrifugation (9,000 rpm for 10 min). The supernate was collected, centrifugation was repeated, and then the supernate was collected again. Serum from young rats is designated “young rat serum” (YRS), whereas that from aged rats was designated as “old rat serum” (ORS).

### RNAi (RNA interference)

The siRNA oligonucleotides were synthesized by Genepharma Co., Ltd. (Shanghai, China). The effective sequence used for the specific silencing of β-catenin was 5′-CACCTCCCAAGTCCTTTAT-3′. The siRNA sequence was named by si-β-catenin. The non-silencing control siRNA is an irrelevant siRNA with random nucleotides 5′-TTCTCCGAACGTGTCACGT-3′ and is not homologous to any sequences found in the gene bank. Transfection was carried out according to the manufacturer's protocol (Qiagen Inc., Valencia, CA).

### Treatment methods of MSCs

There were four groups in the present study. In the YRS or ORS groups, MSCs were cultured for 36 h in DMEM containing 20% YRS or ORS. In the ORS + DKK1 groups, 100 ng/mL Dickkopf-1 (DKK1, R&D Systems, USA) was directly incubated in DMEM containing 20% ORS for 36 h. In ORS + si-β-catenin group, the cells were first transfected with si-β-catenin for 12 h, and then 20% ORS was further added in the culture medium for 36 h.

### SA-β-Gal staining

Senescence-associated β-galactosidase (SA-β-gal) staining was performed using a SA-β-gal staining kit (Genmed Scientifics Inc., China) following the manufacturer's protocol. The treatment methods for the MSCs in each group were the same as described above. The cells were then fixed in 4% (v/v) formaldehyde for 5 min and were stained with SA-β-gal–staining solution at pH 6.0 for 12 h. The SA-β-gal–positive cells exhibited a blue color. The number of positive cells was counted under a phase-contrast microscope. The experiment was repeated five times in each group.

### ROS staining

ROS staining was performed using an ROS staining kit (Genmed, USA) following the manufacturer's protocol. After cultured in each group according to the above treatment methods, the cells were washed three times in PBS and incubated in ROS staining solution (DCFH-DA) at 37°C for 20 min. After washing, the nuclei were counterstained with Hoechst 33342. The cells were observed using a fluorescence microscope. To quantify the ROS level, the DCFH fluorescence intensity in the cells was detected by flow cytometer (Calibur, BD Biosciences, USA) at an excitation wavelength of 488 nm and an emission wavelength of 525 nm. Experiments were repeated three times.

### MTT assay

Assessment of cell proliferation ability was performed according to MTT [3-(4,5-dimethylthiazol-2-yl)-2,5-diphenyl-2H-tetrazolium bromide] assay. The cells were trypsinized and resuspended. Then, the cells were seeded in 96-well plates at a density of 3000–4000 cells per well. The treatment method for the MSCs in each group was the same as described above, and each group had five parallel wells. At the test culture period at Days 1, 2, 3, 4, 5, 6, and 7, the cells were incubated with 5 mg/mL of MTT (Sigma, USA) for the last 4 h. The medium was then removed and formazan salts were dissolved with 150 µL of dimethylsulfoxide (DMSO, Sigma, USA). To establish a growth curve for the cell, the absorbance values were determined at 570 nm with an ELISA reader. The experiment was repeated five times in each group.

### Acridine orange/ethidium bromide (AO/EB) staining

AO/EBapoptotic staining was used to detect MSC apoptosis. The treatment method of the MSCs in each group was the same as that previously described. To increase the level of oxidative stress, the cells were further exposed to 100 µmol/L H_2_O_2_ for 1 h. The cells were then washed three times in PBS at room temperature. The 80 µL AO/EB cocktail (Solomon Bio-Sci & Tech Co, China) was added to the culture plates for 30 min. The cells were examined by fluorescence microscopy (Nikon Eclipse E800 microscope, Japan). The results were documented as previously described [32]. Viable cells stained only with AO were bright green with intact structures, whereas cells in early apoptosis showed bright green nuclear staining. Late apoptotic cells stained with AO_LOW_ and EB were red-orange with condensed chromatin. To determine the apoptotic index, the number of apoptotic cells was divided by the total number of counted cells and multiplied by 100% to calculate the percentage. The experiment was repeated four times in each group.

### Reverse transcription - PCR analysis

Total RNA was extracted with Trizol (Invitrogen, USA) reagent from cells. The RNA was spectrophotometrically quantified at 260 nm. The experiment was performed with an RNA PCR kit (TAKARA, Japan) according to the manufacturer's protocol. The PCR reactions for c-myc, p16^INK4a^, p53, p21, and β-actin mRNA (94°C for 30 s, 54°C for 45 s, 72°C for 1 min, 35 cycles) were carried out using the following forward and reverse primers: c-mycFwd (5′-CCTACCCTCTCAACG ACAGC-3′) and c-mycRev (5′-CTCTAGCCTTTTGCCAGGAG-3′). p16^INK4a^Fwd (5′-ACCAAACGCCCCGAACA-3′) and p16^INK4a^Rev (5′-GAGAGCTGCCACTTT GACGT-3′); p53Fwd (5′-ACCATGAGCGCTGCTCAGAT-3′) and p53Rev (5′-AGTTGCAAACCAGACCTCAG-3′); p21Fwd (5′-TGAATGAAGGCTAAGGCA GAAGA-3′) and p21Rev (5′-AGGCAGACCAGCCTAACAGATT-3′); and β-actinFwd (5′-AAGAGAGGCATCCTCACCCT-3′) and β-actinRev (5′-TACATG GCTGGGGTGTTGAA-3′). The PCR products were analyzed on 1.5% agarose gel and visualized under UV light following ethidium bromide staining. Quantitative data were expressed by normalizing the densitometric units to β-actin (internal control).

### Immunofluorescence

To examine the expression of β-catenin in young or old rat MSCs, the primary MSCs were isolated from SD rats aged 12–14 or 64–68 weeks as previously described. The cells were then seeded onto 35 mm dishes and cultured in DMEM without serum for 24 h. Thereafter, the cells were fixed with 4% (v/v) formaldehyde for 10 min. After washing with PBS, the cells were blocked with 10% BSA, and incubated at 37°C for 1 h with rabbit anti-rat β-catenin (1∶200, Cell Signaling, USA) and mouse anti-rat GD2 (1∶200, Imgenex, USA). Then, the cells were washed and incubated in the dark for 1 h at 37°C with goat anti-rabbit (cy3)-conjugated antibodies (1∶300, ICN Cappel, USA) and goat anti-mouse FITC-conjugated antibodies (1∶300, Dako, USA). After washing, the nuclei were counterstained with DAPI.

For evaluating the effect of ORS on the expression of β-catenin, GSK-3β, γ-H2A.X, and p53, the young MSCs were incubated with β-catenin (1∶200, Cell Signaling, USA), GSK-3β (1∶200, Santa Cruz, USA), γ-H2A.X (1∶100, Santa Cruz, USA), or p53 (1∶200, Santa Cruz, USA) polyclonal rabbit anti-rat antibodies. Thereafter, the cells were washed and incubated with goat anti-rabbit (cy3)-conjugated antibodies or FITC-conjugated antibodies (1∶300, ICN Cappel, USA). DAPI was used to visualize nuclei. After washing and being mounted, the cells were examined under a fluorescence microscope.

### Western blot analysis

To assay the β-catenin protein, cytoplasmic and nuclear proteins from cultured cells were prepared using NE-PER nuclear and cytoplasmic extraction reagents (Pierce Chemical Company, USA), respectively. β-Actin and β-tubulin were used as the internal control for the cytoplasmic and nuclear proteins. To assay GSK-3β, γ-H2A.X, and p53, the total cellular protein was extracted through the following methods: The different MSC treatment groups were washed in cold-buffered PBS and were then lysed in RIPA buffer (150 mM NaCl, 1% Triton X-100, 0.5% NaDOD, 0.1% SDS, and 50 mM Tris, pH 8.0). After centrifugation (12,000 rpm, 5 min) at 4°C, the protein supernate was transferred into new tubes. The protein concentration of the samples was determined with a bicinchoninic acid protein assay (Pierce, USA). A 40 µg sample of the total protein was resolved using 12.5% SDS-PAGE and transferred onto polyvinylidene difluoride (PVDF, Millipore, USA) membranes. The membranes were blocked with 5% nonfat milk at room temperature for 1 h in Tris-buffered saline containing Tween 20 (TBST). Primary antibodies to detect β-catenin, GSK-3β, γ-H2A.X, p53, β-actin (Biovision, USA), or β-tubulin (Cell Signaling, USA) were incubated overnight with the membranes at 4°C. Membranes were incubated with horseradish peroxidase (HRP)-conjugated anti-rabbit secondary antibodies (1∶2000 dilution, Dako, USA), and proteins were detected by enhanced chemiluminescence (ECL) (Amersham Biosciences Corp, USA). β-Actin was used as the internal control to normalize the loading materials.

### Statistical Analysis

All experiments were performed at least in triplicate. All data are presented as mean ± standard deviation (SD) of the replicates. Significance testing was performed using one-way ANOVA to compare data from different experimental groups. A *P*<0.05 was considered statistically significant.

## Results

### Effects of ORS on the MSCs senescence

**Figure 1:**
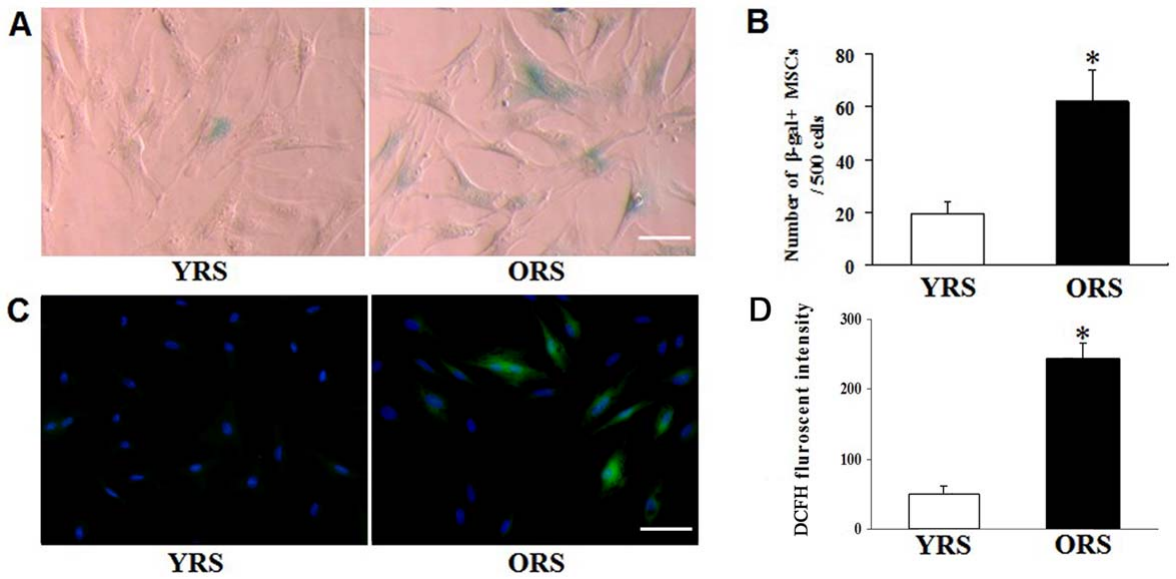
Effects of ORS on SA-β-gal expression and ROS fluorescence level in MSCs. (A) SA-β-gal staining. Compared with the YRS group, the number of SA-β-gal–positive cells in the ORS group was clearly increased, and those SA-β-gal–positive cells show flat and enlarged cell shape. Scale bar  = 25 µm. (B) Quantification of SA-β-gal–positive cells. The total number of SA-β-gal–positive cells among 500 random cells was counted using phase-contrast microscopy. The results show that the number of SA-β-gal–positive MSCs/500 cells in the ORS group was significantly higher than that in the YRS group(*P<0.01; *n* = 5). (C) ROS staining. In the ORS group, more ROS–stained cells were observed through a fluorescence microscope. Green, ROS staining; blue, Hoechst 33342 staining. Scale bar  = 25 µm. (D) Quantification assays of the ROS level. Intracellular ROS generation was determined by DCFH fluorescence. The DCFH fluorescence intensity in the ORS group was evidently higher compared with the YRS group (* *P*<0.01; *n* = 3).

SA-β-gal staining was used to examine the effects of ORS on MSC senescence. The MSCs which were not specially illustrated in our study were derived from SD rats 12–14 weeks old, and passage 3–5 cells were used. After culturing with ORS for 36 h, the number of SA-β-gal–positive cells obviously increased (Figure 1A). The cell count revealed that the number of SA-β-gal–positive cells was significantly different between the ORS group (62.0±11.7) and the YRS group (19.4±4.8; *P*<0.01) (Figure 1B). ROS staining shows that the number of ROS–stained cells and the DCFH fluorescent level of the cells were higher in the ORS group compared with that in the YRS group (Figure 1C–D). These results suggested that ORS promoted senescence of MSCs.

### Effect of ORS on MSCs proliferation

**Figure 2:**
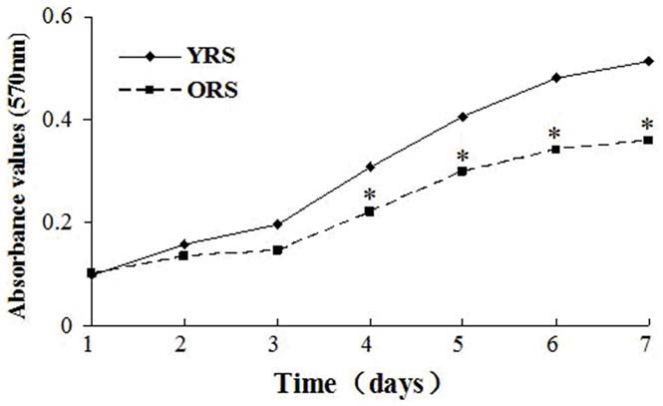
Cell proliferation curves of MSCs. MTT assay show that the proliferation of the cells was not significantly different between the YRS and ORS groups at 1 and 2 days. The absorbance in the ORS group were significantly lower than that in the YRS group from 4 to 7 days (*P<0.05; *n* = 5).

The proliferation of MSCs was measured with an MTT assay. The results indicate that MSC proliferation is inhibited by the ORS culture, and the absorbance values in the ORS group were markedly decreased compared with that in the YRS group (*P*<0.05, Figure 2).

### Effects of ORS on MSCs survival

**Figure 3:**
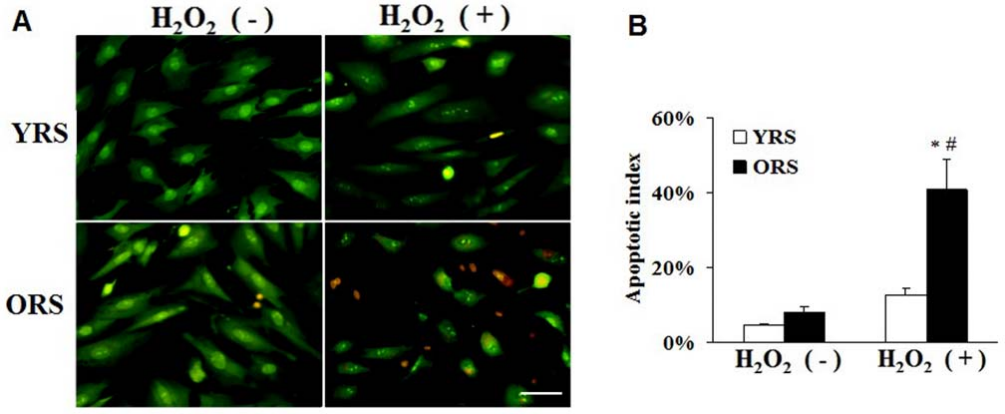
Effects of ORS on the survival and apoptosis of MSCs. (A) AO/EB staining. Most cells in the YRS group showed normal cell morphologies. In the ORS group, a small number of apoptotic cells (green- or red-orange–stained condensation nuclei) were observed. After 100 µmol/L H_2_O_2_ treatment for 1 h, the number of apoptotic cells in the YRS group remained low. However, in the ORS group, the apoptotic and necrotic cells (red-orange–stained nucleus with normal structure) was clearly increased. Scale bar  = 25 µm. (B) MSC apoptotic index. After treatment with 100 µmol/L H_2_O_2_ in the ORS group, the apoptotic index of the cells significantly increased compared with that in the YRS group (*P<0.01) or that in the ORS group without H_2_O_2_ treatment (^#^ *P*<0.01; *n* = 4).

After treatment with YRS or ORS for 36 h, AO/EB staining was used to observe the apoptotic cells. The apoptotic cells had green or red-orange stained condensation nucleus. In the YRS group, no obvious apoptosis was observed, whereas a few apoptotic cells were observed in the ORS group. After treatment with 100 µmol^/^L H_2_O_2_ for 1 h, apoptosis was still not obvious in the YRS group, but in the ORS group, the number of apoptotic cells obviously increased (Figure 3A). The cell count shows that the apoptotic index in the ORS group after H_2_O_2_ treatment (40.9%±7.8%) was significantly higher than that in the YRS group (12.6%±2.1%, *P*<0.01) or that in the ORS group without H_2_O_2_ treatment (8.2%±1.5%, *P*<0.01) (Figure 3B). The results indicated that the ORS could inhibite the survival of MSCs.

### The expression of β-catenin in the young or old rat MSCs

**Figure 4:**
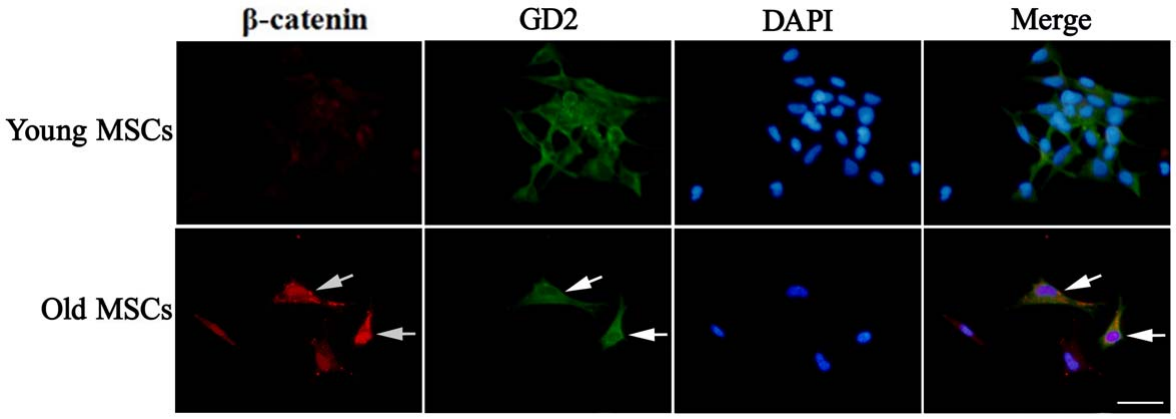
β-Catenin and GD2 expression in MSCs from young and old rats. The immunofluorescence staining results show that the β-catenin expression is very weak in the young GD2–positive cells, but clearly increased in old GD2–positive cells. The arrow indicates the old cells co-expressed with β-catenin and GD2. Red, β-catenin; green, GD2; blue, DAPI. Scale bar  = 25 µm.

To identify the activity of Wnt/β-catenin signaling in MSCs of old rats, the β-catenin expression in the MSCs of young rats (12–14 weeks old) and of old rats (64–68 weeks old) was examined by immunofluorescence. The disialoganglioside (GD2) is a new marker of MSCs and can express on primary MSCs, and the MSCs are the only cells that express this marker in bone marrow [33], so GD2 was used to mark primary MSCs. The results showed that β-catenin expression clearly increased in the GD2–positive cells of the old rats compared with that of the young rats (Figure 4). The results indicated the Wnt/β-catenin signaling was elevated in old rat MSCs.

### ORS promoted the nuclear accumulation of β-catenin in MSCs

**Figure 5:**
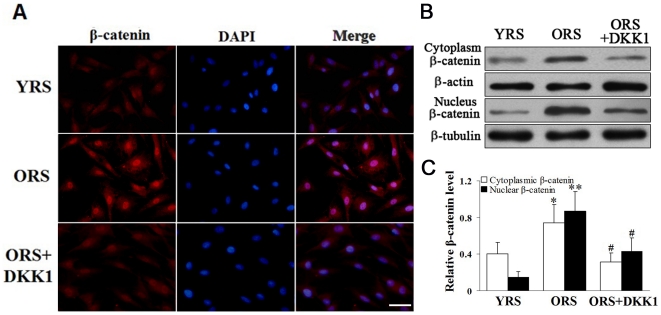
Effect of ORS on β-catenin expression. (A) Immunofluorescence staining of β-catenin. In the ORS group, there was a clear increase in nuclear β-catenin expression. After treatment with 100 ng/mL DKK1, the nuclear β-catenin expression decreased. Scale bar  = 25 µm. (B) Western blot analysis of the cytoplasmic and nuclear β-catenin. β-Actin was used as the internal control for the cytoplasmic proteins, whereas β-tubulin was used as the internal control for the nuclear proteins. (C) Quantification of cytoplasmic and nuclear β-catenin protein levels. An obvious increase in β-catenin protein level was detected in the cytoplasm (*P<0.05) and nuclei (**P<0.01) of the cells in the ORS group compared with that in the YRS group. In the ORS + DKK1 group, the β-catenin levels were clearly decreased compared with that in the ORS group (**^#^** *P*<0.01; *n* = 5).

To investigate the effects of ORS on Wnt/β-catenin signaling, we examined the expression of β-catenin in MSCs by immunofluorescence staining and western blot analysis. The expression of cytoplasmic and nuclear β-catenin increased in the ORS group compared with that in the YRS group, and the increase in nuclear β-catenin expression was more pronounced in the ORS group. After treatment with 100 ng/mL DKK1, nuclear β-catenin expression markedly decreased (Figure 5). To further evaluate the effects of ORS on the nuclear translocation of β-catenin, the expression of β-catenin was examined at different time points during the ORS culture. The results demonstrated that the nuclear translocation of β-catenin could be promoted by ORS (Supporting Information, Figure S1).

### ORS reduced GSK-3β expression in MSCs

**Figure 6:**
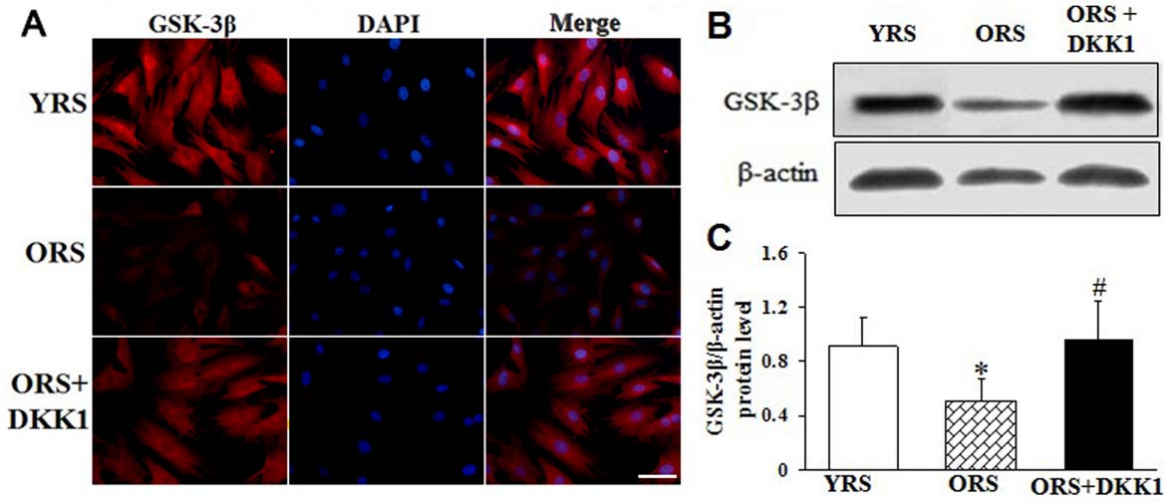
Effect of ORS on GSK-3β expression. (A) Immunofluorescence staining of GSK-3β. Compared with that in the YRS group, GSK-3β expression was weak in the ORS group. However, after treatment with 100 ng/mL DKK1, the GSK-3β expression increased. Red, GSK-3β; blue, DAPI. Scale bar  = 25 µm. (B) Western blot analysis of GSK-3β expression. β-Actin was used as the internal control. (C) Quantification of GSK-3β protein levels. GSK-3β protein levels were significantly decreased in the ORS group compared with the YRS group (*P<0.05) and the ORS + DKK1 group (**^#^** *P*<0.05; *n* = 5).

GSK-3β is a key enzyme in negatively regulating Wnt/β-catenin signaling. The effect of ORS on Wnt/β-catenin signaling was further studied by determining GSK-3β expression. Compared with the YRS group, GSK-3β expression was evidently decreased in the ORS group (*P*<0.05). However, after treatment with DKK1, GSK-3β expression was significantly increased (*P*<0.05, Figure 6).

### ORS promoted c-myc expression in MSCs

**Figure 7:**
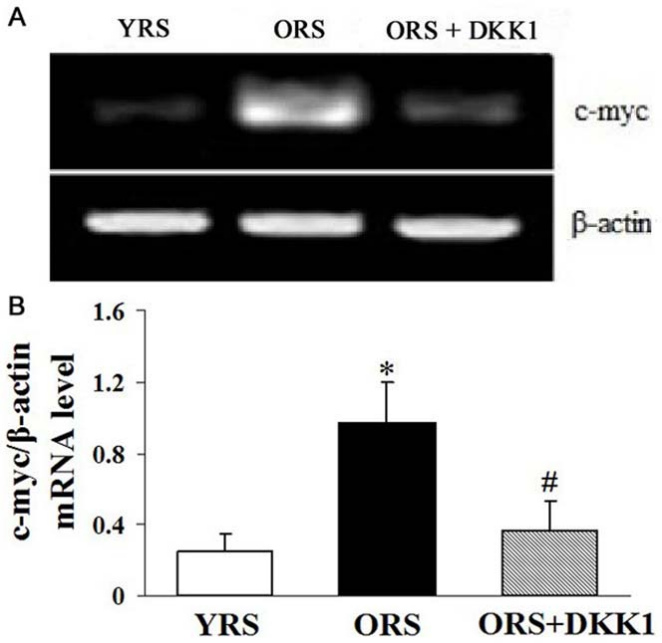
Effect of ORS on c-myc expression. (A) RT-PCR analysis of the expression of c-myc. β-Actin was used as an internal control. (B) Quantification of c-myc mRNA expression level. The expression of c-myc mRNA in the ORS group was markedly increased than that in the YRS group (*P<0.01). However, after treatment with DKK1 to inhibitor the Wnt/β-catenin signaling in ORS, the c-myc mRNA level was significantly decreased compared with that in the ORS groups (^#^ *P*<0.01; *n* = 5).

To further examine the expression of target gene of Wnt/β-catenin signaling, we detected the expression of c-myc by RT-PCR. The expression of c-myc in the ORS group is significantly higher than that in the YRS group (*P*<0.01). After treatment with DKK1, c-myc expression was significantly decreased (*P*<0.01, Figure 7). These data further indicated that the Wnt/β-catenin signaling could be activated by ORS.

### Effects of Wnt/β-catenin signaling on MSCs senescence

**Figure 8:**
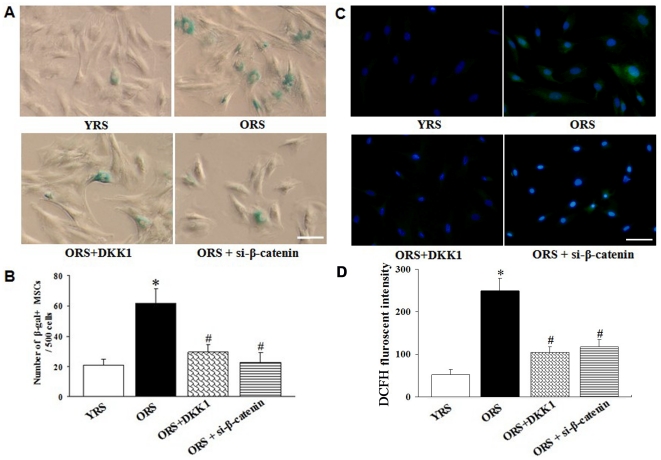
Effect of Wnt/β-catenin signaling on MSC senescence. (A) SA-β-gal staining. After treatment with DKK1 and si-β-catenin in ORS, the number of SA-β-gal–positive cells decreased. Scale bar  = 25 µm. (B) Quantification of SA-β-gal–staining cells. The number of SA-β-gal–positive cells significantly decreased in the ORS + DKK1 group and in the ORS + si-β-catenin group compared with that in the ORS group (**^#^** *P*<0.01). *P<0.01 versus the YRS group (*n* = 5). **(C)** ROS staining**.** Scale bar  = 25 µm. (D) Quantification of ROS level. The fluorescence intensity of DCFH was significantly lower in the ORS + DKK1 group or ORS + si-β-catenin group compared with that in the ORS group (**^#^** *P*<0.01). *P<0.01 versus YRS group (*n* = 3).

To explore the effects of Wnt/β-catenin signaling on MSCs senescence, we examined cellular senescence after modulating Wnt/β-catenin signaling through SA-β-gal staining. To examine better the relationship between Wnt/β-catenin signaling and cellular senescence in the YRS group, different concentrations of Wnt 3a were added into the culture medium. The results suggest that only treatment with high Wnt 3a concentrations (100 ng/mL) significantly increases the number of SA-β-gal–positive cells (Supporting Information, Figure S2). In the ORS group, siRNA that specifically silences β-catenin mRNA was designed and synthesized to inhibit intracellular Wnt/β-catenin signaling further (Supporting Information, Figure S3). The results show that after Wnt/β-catenin signaling was inhibited with DKK1 or si-β-catenin, the number of SA-β-gal–positive cells in the two groups (29.2±4.7 and 22.6±6.5) were significantly decreased compared with that in the ORS group (61.6±9.6, *P*<0.01) (Figure 8A–B).

ROS staining showed that after Wnt/β-catenin signaling was inhibited by DKK1 or si-β-catenin in ORS, the level of DCFH fluorescence in the MSCs clearly decreased (Figure 8C–D).

### Effect of Wnt/β-catenin signaling on MSCs proliferation

**Figure 9:**
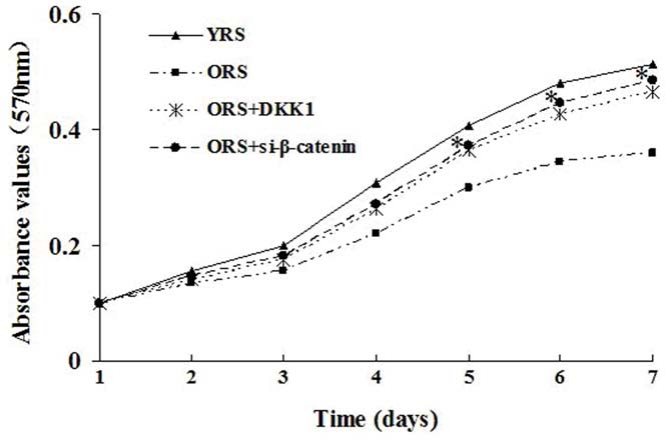
MSC proliferation curves. MTT assay showed there was no significant difference in the proliferation of cells within 1, 2, and 3 days. From days 5 to 7, the absorbance values in the ORS + DKK1 and ORS + si-β-catenin groups were significantly higher compared with that in the ORS group (^*^ *P*<0.05: *n* = 5).

MTT analysis shows that after treatment with DKK1 or si-β-catenin in ORS, the absorbance values of the two groups were significantly higher than those of the ORS group (*P*<0.05, Figure 9).

### Effects of Wnt/β-catenin signaling on MSCs survival

**Figure 10:**
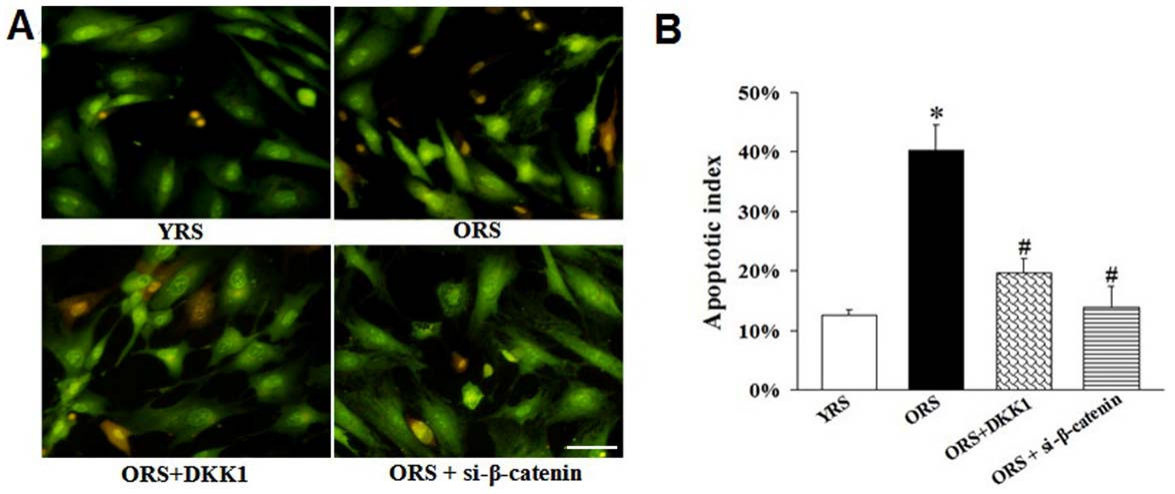
Effects of Wnt/β-catenin signaling on survival and apoptosis of MSCs. (A) AO/EB staining. In the ORS+ DKK1 and ORS + si-β-catenin group, the number of apoptotic or necrotic cells decreased. Scale bar  = 25 µm. (B) The apoptotic index of the MSCs. The apoptotic indices of the ORS + DKK1 and the ORS + si-β-catenin groups significantly decreased compared with that in the ORS group (**^#^** *P*<0.01). *P<0.01 versus YRS group (*n* = 4).

After Wnt/β-catenin signaling was inhibited with 100 ng/mL DKK1 or si-β-catenin in ORS, the number of apoptotic cells decreased (Figure 10A). The apoptotic indices in the ORS + DKK1 group (19.7%±2.4%) and ORS + si-β-catenin group (12.3%±2.5%) were significantly decreased compared with that in the ORS group (40.3%±4.4%, *P*<0.01) (Figure 10B). The flow cytometry analysis of Annexin V/PI staining also showed that the apoptotic index in the ORS group increased more than that in the YRS group. After treatment with DKK1 or si-β-catenin in ORS, the apoptotic index significantly decreased (Supporting Information, Figure S4).

### Activated Wnt/β-catenin signaling induced DDR in MSC

**Figure 11:**
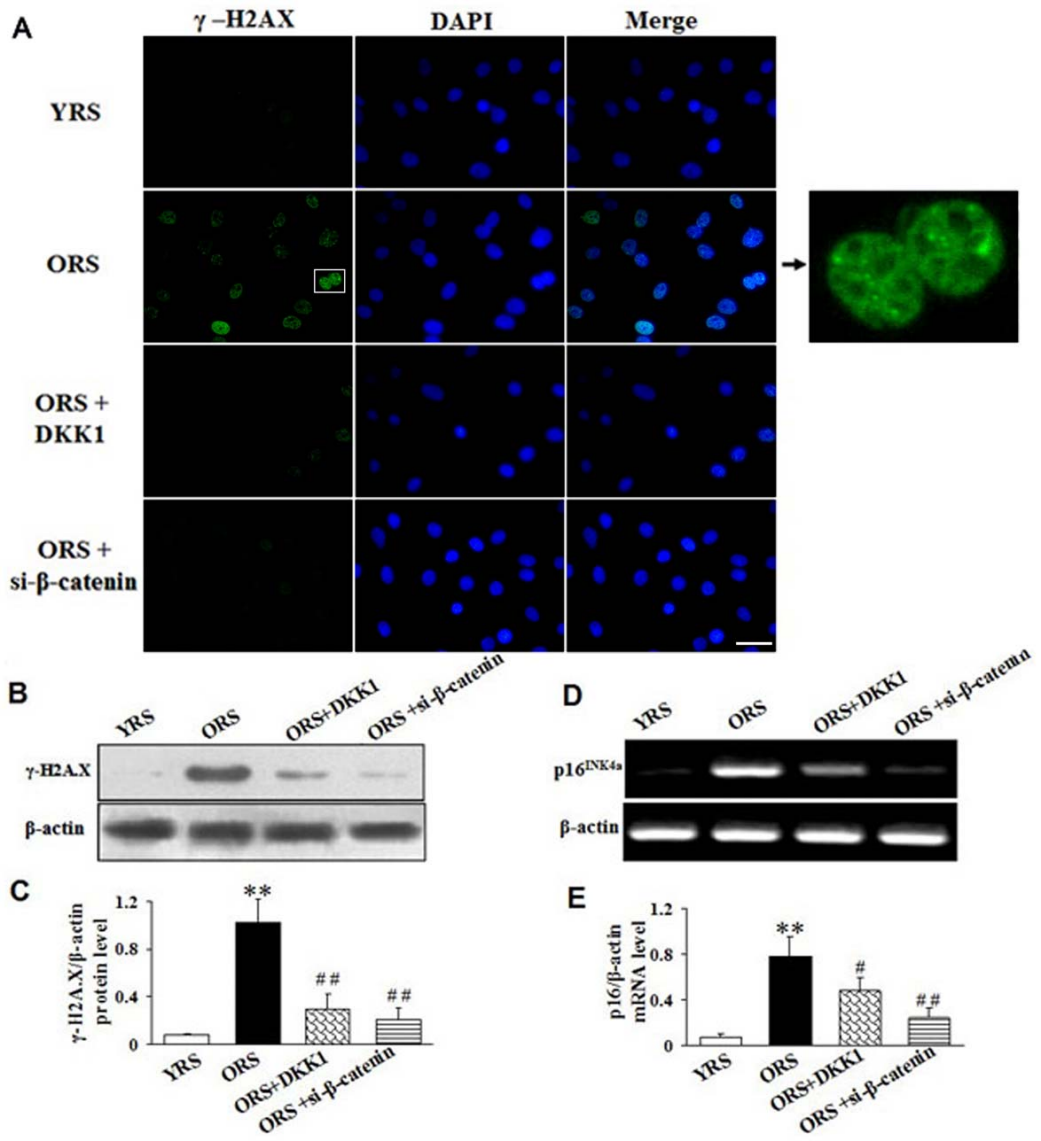
Effects of Wnt/β-catenin signaling on γ-H2A.X and p16^INK4a^ expression. (A) Immunofluorescence staining of γ-H2A.X. The expression of γ-H2A.X was hardly detected in the YRS group, whereas the number of γ-H2A.X–positive cells and γ-H2A.X expression level were markedly increased in the ORS group. In the ORS + DKK1 and ORS + si-β-catenin groups, the number of γ-H2A.X–positive cells and γ-H2A.X expression levels were clearly decreased. The pictures on the right are magnified images of the white box. γ-H2A.X expression is indicated by a stippled appearance in the nucleus. Green, γ-H2A.X; blue, DAPI. Scale bar  = 25 µm. (B) Western blot analysis of γ-H2A.X expression. β-Actin was used as the internal control. (C) Quantification of γ-H2A.X protein expression levels. γ-H2A.X expression was significantly increased in the ORS groups compared with that in the YRS groups (**P<0.01). After treatment with DKK1 or si-β-catenin in ORS, γ-H2A.X expression was markedly decreased compared with the ORS group (**^##^** *P*<0.01; *n* = 5). (D) RT-PCR analysis of p16^INK4a^ expression. β-Actin was used as the internal control. (E) Quantification of p16^INK4a^ mRNA expression level. p16^INK4a^ expression in the ORS group markedly increased compared with that in the YRS group (**P<0.01). However, in the ORS + DKK1 and the ORS + si-β-catenin groups, the p16^INK4a^ mRNA levels were significantly decreased compared with the ORS groups (^#^ *P*<0.05 or ^# #^ *P*<0.01; *n* = 5).

To explore whether Wnt/β-catenin signaling have any effects on DDR, we examined the expression of γ-H2A.X, a sensitive marker for the formation of DNA damage foci. The results suggest that compared with the YRS group, the number of γ-H2A.X–positive cells and the γ-H2A.X expression level were markedly increased in the ORS group. After treatment with DKK1 or si-β-catenin, the number of γ-H2A.X–positive cells and the γ-H2A.X level markedly decreased (*P*<0.01, Figure 11A–C). A recent study suggested that DDR induces cell aging by directly activating p16^INK4a^ expression [28]. Therefore, we examined the expression of p16^INK4a^ by RT-PCR. The RT-PCR results show that p16^INK4a^ expression was significantly increased in the ORS groups compared with that in the YRS groups. In the ORS + DKK1 group and the ORS + si-β-catenin group, p16^INK4a^ expression clearly decreased (*P*<0.05 or *P*<0.01, Figure 11D–E).

### Activated Wnt/β-catenin signaling promoted expression of p53 and p21 in MSCs

**Figure 12:**
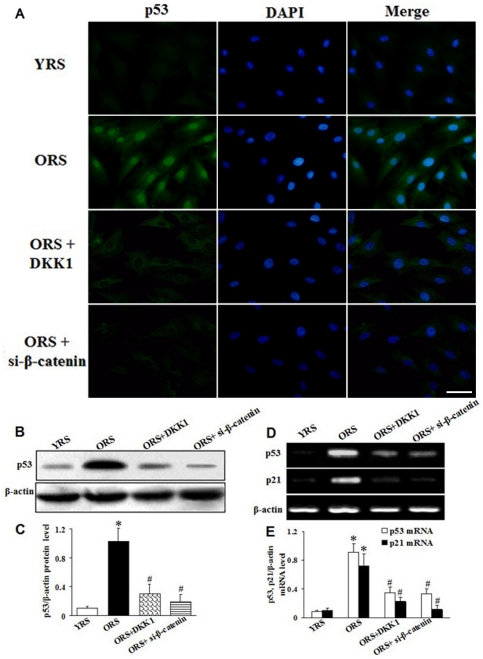
Effects of Wnt/β-catenin signaling on p53 and p21 expression. (A) Immunofluorescence staining of p53. p53 expression was hardly detected in the YRS group, whereas the ORS group showed an obvious increase in p53 expression. p53 expression was decreased after treatment with DKK1 or si-β-catenin to inhibit Wnt/β-catenin signaling. Green, p53; blue, DAPI. Scale bar  = 25 µm. (B) Western blot analysis of p53 protein expression. β-Actin was used as the internal control. (C) Quantification of p53 protein levels. The p53 expression level was clearly higher in the ORS group than that in the YRS group (*P<0.01). In the ORS + DKK1 and ORS + si-β-catenin groups, the p53 expression level was evidently lower than in the ORS group (**^#^** *P*<0.01; *n* = 5). (D) The expression of p53 and p21 were assessed using RT-PCR. β-Actin was used as the internal control. (E) Quantification of p53 and p21 mRNA level, normalized with β-actin mRNA level. The results show there was a clear upregulation of p53 and p21 mRNA expression in the ORS group compared with that in the YRS group (*P<0.01). After treatment with DKK1 or si-β-catenin to inhibit Wnt/β-catenin signaling in ORS, p53 and p21 expression was significantly decreased compared with that in the ORS group (**^#^** *P*<0.01; *n* = 5).

To investigate the effects of Wnt/β-catenin signaling on the p53/p21 pathway, we first examined p53 expression through immunofluorescence and western blot analysis. In the YRS group, p53 expression was very weak, whereas the ORS group showed a clear increase in p53 expression. Compared with the ORS group, p53 expression was substantially inhibited in the ORS + DKK1 or ORS + si-β-catenin group (Figure 12A–C). The target gene of p53, p21, plays an important role in cell aging induced by p53. Therefore, we further tested the expression of p53 and p21 through RT-PCR. The results show that p53 and p21 expression were obviously increased in the ORS group compared with that in the YRS group (*P*<0.01, Figure 12D–F). However, after treatment with DKK1 or si-β-catenin in ORS, p53 and p21 were significantly decreased compared with that in the ORS group (*P*<0.01, Figure 12D–F).

## Discussion

Increasing studies have demonstrated the importance of extrinsic cellular factors on the aging of adult stem cells. Aged mouse spermatogonial stem cells have been transplanted into young recipient hosts for over three years without any decline in function [34]. Serum from old mice markedly induces embryonic stem cell dysfunction [13]. However, the effects of the aged environment on MSC senescence and function have not yet been reported. In the present study, the young and the old systemic milieu were mimicked by adding 20% YRS and ORS into the culture medium respectively. The results show that the ORS culture clearly promoted senescence and ROS production in the MSCs compared with those cultured with YRS. The proliferation and survival ability of the MSCs were also significantly inhibited in the ORS group compared with that in the YS group. Therefore, ORS induces MSC senescence, as well as inhibit their proliferation and survival ability.

Studies have demonstrated that some signaling pathways also have important effects on the cell senescence induced by an aged systemic environment [10], [35], [36]. Recent papers [17], [20] have shown that Wnt/β-catenin signaling is elevated in aged tissue and in a mouse model of accelerated aging. This elevated Wnt/β-catenin signaling may contribute to cell senescence. Our study shows that β-catenin expression increases in primary MSCs of old rat. ORS treatment promotes the nuclear accumulation of β-catenin in young rat MSCs, which is inhibited by DKK1. These results indicate that the Wnt/β-catenin signaling in MSCs can be activated by the aged system enviroment and the presence of soluble factors in ORS that mediate the activity of Wnt/β-catenin signaling. Research by Binet has shown that senescent cells secrete more Wnt 16 b, a member of the Wnt family of secreted proteins [37]. However, whether Wnt 16 b is the activator of Wnt/β-catenin signaling in ORS or some other “Wnt-like substance” requires further verification. To explore further the relationship between MSC senescence and the activity of Wnt/β-catenin signaling, we examined the changes in the MSCs by modulating Wnt/β-catenin signaling. In the YRS group, only excessive Wnt/β-catenin signaling activation could induce MSC senescence. In the ORS group, after treatment with DKK1 or si-β-catenin, the number of SA-β-gal–positive cells was significantly decreased, and the proliferation and survival ability were evidently enhanced. Taken together, activated Wnt/β-catenin signaling is an important mediator of MSC aging induced by ORS.

Based on these results, we explored the mechanisms of Wnt/β-catenin signaling on MSC aging. DDR may induce cell senescence [38]. Recent data have shown that DDR can be found in almost all of the organs of aged mice [39]. The DDR induces cell senescence through the p16^INK4a^ gene [27], [28] or the p53/p21 pathway [40], [41]. However, whether activated Wnt/β-catenin signaling promotes cellular senescence through the DDR and p53/p21 pathway remains unclear. In the present study, a clear increase in γ-H2A.X, p16^INK4a^, p53, and p21 expression was observed in the ORS group compared with that in the YRS group. After the 100 ng/mL DKK1 and si-β-catenin treatment, the expression of γ-H2A.X, p16^INK4a^, p53, and p21 were substantially decreased. These results imply that the activation of Wnt/β-catenin signaling induces MSC aging though the DDR and p53/p21 pathways. One recent published report has also shown that the consequences of persistent Wnt1-induced epithelial cell senescence and progressive hair loss in transgenic mice. The number of γ-H2A.X–positive cells increased in the hair follicles [42]. The activated c-myc, a target gene of Wnt/β-catenin signaling, induces cellular senescence by promoting p16^INK4a^ expression [43], which is consistent with the results of the present study. Therefore, there is a correlation between Wnt/β-catenin signaling and the DDR and p53/p21 pathway, which are potential molecular targets for delaying stem cell aging in aged systemic milieus.

In conclusion, ORS can induce the senescence of adult MSCs, as well as inhibit their proliferation and survival. Wnt/β-catenin signaling plays a critical role in the aging of MSCs induced by ORS. The DNA damage response and p53/p21 pathway may be the main mediators of MSC aging induced by excessive Wnt/β-catenin signaling. Further deciphering the mechanisms of Wnt/β-catenin signaling involved in stem cell aging will help improve transplantation efficacy of stem cells for older persons.

## Supporting Information

Figure S1 **Effect of ORS on β-catenin expression at different time points.** (A) Immunofluorescence staining of β-catenin. Cytoplasmic β-catenin expression after culturing with ORS for 12 h was obviously increased compared with that in the cells cultured with ORS for 0 h. When the cells were cultured with ORS for 24 h, the expression of cytoplasm β-catenin decreased and the expression of nucleus β-catenin increased. After culturing with ORS for 36 h, the expression of nucleus β-catenin further increased. Scale bar  = 25 µm. (B) Western blot analysis of cytoplasmic and nuclear β-catenin. β-Actin was used as the internal control for cytoplasmic proteins, whereas β-tubulin was used as the internal control for nuclear proteins. (C) Quantification of cytoplasmic and nuclear β-catenin protein levels. Compared with those cultured with ORS for 0 h, an obvious increase in β-catenin protein level was detected in the cytoplasm (*P<0.01) of MSCs cultured from 12 h to 36 h. However, after culturing for 36 h, the cytoplasm β-catenin protein level significantly decreased compared with that in the MSCs cultured for 12 h (^#^ *P*<0.05). The nuclear β-catenin protein level gradually increased in the cells cultured with ORS from 0 to 36 h. $ *P*<0.05 versus 0 h, $$ *P*<0.01 versus 0 h, † *P*<0.01 versus 12 h, § *P*<0.05 versus 24 h, *n* = 4.
(TIF)

Figure S2 **Effects of different concentrations of Wnt 3a on MSC senescence.** (A) SA-β-gal staining. Similar to the YRS group, after stimulation with 10 or 50 ng/mL Wnt 3a (R&D Systems, USA) in YRS for 36 h, only a small number of SA-β-gal–positive cells were observed. However, in the YRS +100 ng/mL Wnt 3a group, the number of SA-β-gal–positive cells obviously increased. Scale bar  = 25 µm. (B) Quantification of SA-β-gal–positive cells. The number of SA-β-gal–positive cells was not significantly increase in the YRS +10 ng/mL Wnt 3a and the YRS +50 ng/mL Wnt 3a groups compared with that in the YRS group (22.4±5.5 or 24.6±8.7 vs. 20.8±4.2. *P* > 0.1). However in the YRS +100 ng/mL Wnt 3a group, the number of SA-β-gal–positive cells (56.2±12.1) was significantly increased compared with that in the YRS group (*P<0.01). *n* = 5.
(TIF)

Figure S3 **Detection of si-β-catenin transfection efficiency.** (A) Up to 500 ng of FAM-labeled NC-siRNA was transfected into the MSCs for 12 h. Transfection efficiency was detected through phase-contrast and fluorescence microscopy. The results showed that siRNA had entered the cells. Scale bar  = 25 µm. (B) Flow cytometry was also employed to detect the transfection efficiency of siRNA. The results were analyzed with Win MDI29 software, which indicated that siRNA transfection efficiency in MSCs was 88.63%. (C) RT-PCR analysis of β-catenin expression for screening the effective siRNA fragment. Three siRNA oligonucleotide targets were designed and synthesized to silence β-catenin (si-β-catenin1: 5′-GCTGACCAAACTGCTAAAT-3′; si-β-catenin2: 5′-CACCTCCCAAGTCCTTTA T-3′; si-β-catenin3: 5′-GCACCATGCAGAATACAAA-3′). MSCs were incubated with the control siRNA and three si-β-catenin for 12 h. The MSCs were further exposed to ORS for 12 h. β-catenin mRNA expression was examined by RT-PCR. The results show that β-catenin mRNA expression is significantly inhibited by si-β-catenin2 and si-β-catenin3. β-Actin was used as the internal control. The methods for RNA extraction and RT-PCR were the same as described in the Materials and Methods section. β-CateninFwd (5′-ACAGCACCTTCAGCACTCT-3′) and β-cateninRev (5′-AAGTTCTTGGCTATTACGACA-3′). (D) Western blot analysis of β-catenin expression for screening the effective siRNA fragment. After transfection with si-β-catenin for 48 h, the total β-catenin levels were assessed through western blot analysis. β-Actinoligonucleotide was used as the internal control. For the whole-cell extracts, the expression of β-catenin protein is significantly inhibited by si-β-catenin2 and si-β-catenin3. The si-β-catenin2 had a more efficient silencing effect. Thus, the si-β-catenin used in the present study was si-β-catenin2. (E) Immunofluorescence staining of β-catenin. After transfected with si-β-catenin for 48 h, MSCs were fixed with formaldehyde, stained for β-catenin (red) and DAPI (blue), and observed through fluorescence microscopy. The results show that β-catenin expression was significantly decreased after si-β-catenin (si-β-catenin2) transfection in MSCs. Scale bar  = 25 µm.
(TIF)

Figure S4 **Flow cytometry analysis of apoptotic cells stained with Annexin V and PI.** (A) Representative graphs of flow cytometry analysis. To further examine the apoptosis, the apoptotic cells were determined using an Annexin V/PI apoptosis detection kit (Sigma, USA) for flow cytometry (Calibur, BD Biosciences, USA), according to the manufacturer's instructions. Up to 2 × 10^4^ cells for each sample were analyzed using CellQuest software. Every experiment was performed in quadruplicate. (B) The apoptotic index of different groups according to annexin V/PI staining. The apoptotic index clearly increased in the ORS group compared with that in the YRS group (*P<0.01). However, after treatment with DKK1 or si-β-catenin in ORS to inhibit Wnt/β-catenin signaling, the apoptotic index significantly decreased (^#^ *P*<0.01). *n* = 4.
(TIF)
