# Supplementary figures and images for: Wnt/β-Catenin Signaling Induces the Aging of Mesenchymal Stem Cells through the DNA Damage Response and the p53/p21 Pathway
Source: PLoS One. 2011 Jun 21;6(6):e21397. doi: 10.1371/journal.pone.0021397 (PMC3119703; doi:10.1371/journal.pone.0021397)

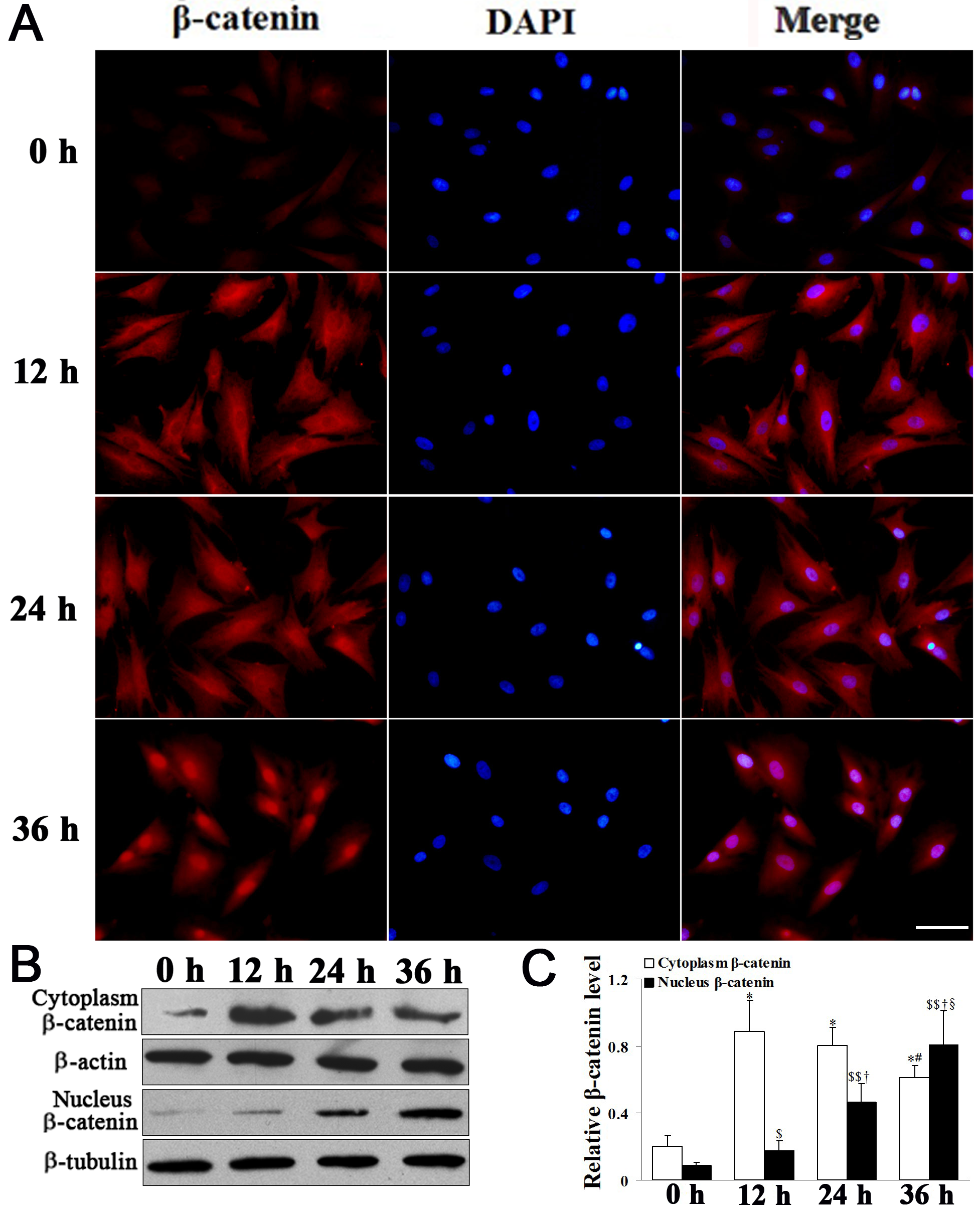

Supplement: Figure S1 — Effect of ORS on β-catenin expression at different time points. (A) Immunofluorescence staining of β-catenin. Cytoplasmic β-catenin expression after culturing with ORS for 12 h was obviously increased compared with that in the cells cultured with ORS for 0 h. When the cells were cultured with ORS for 24 h, the expression of cytoplasm β-catenin decreased and the expression of nucleus β-catenin increased. After culturing with ORS for 36 h, the expression of nucleus β-catenin further increased. Scale bar = 25 µm. (B) Western blot analysis of cytoplasmic and nuclear β-catenin. β-Actin was used as the internal control for cytoplasmic proteins, whereas β-tubulin was used as the internal control for nuclear proteins. (C) Quantification of cytoplasmic and nuclear β-catenin protein levels. Compared with those cultured with ORS for 0 h, an obvious increase in β-catenin protein level was detected in the cytoplasm (*P<0.01) of MSCs cultured from 12 h to 36 h. However, after culturing for 36 h, the cytoplasm β-catenin protein level significantly decreased compared with that in the MSCs cultured for 12 h (# P<0.05). The nuclear β-catenin protein level gradually increased in the cells cultured with ORS from 0 to 36 h. $ P<0.05 versus 0 h, $$ P<0.01 versus 0 h, † P<0.01 versus 12 h, § P<0.05 versus 24 h, n = 4. (TIF) [file pone.0021397.s001.tif]

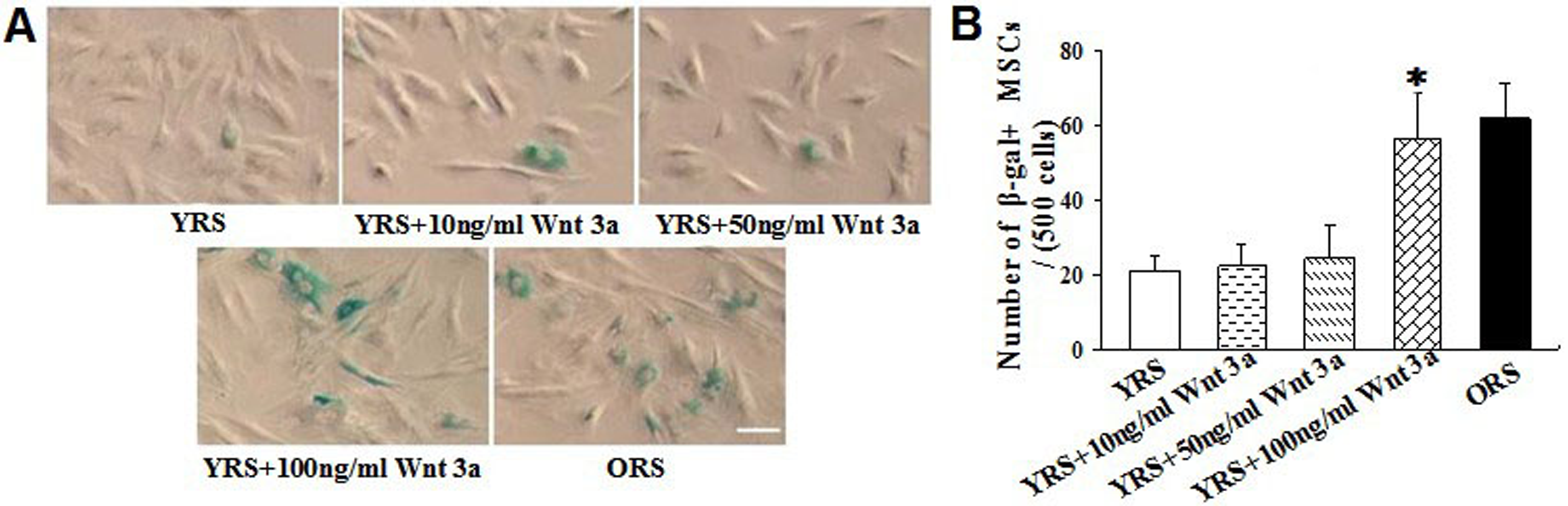

Supplement: Figure S2 — Effects of different concentrations of Wnt 3a on MSC senescence. (A) SA-β-gal staining. Similar to the YRS group, after stimulation with 10 or 50 ng/mL Wnt 3a (R&D Systems, USA) in YRS for 36 h, only a small number of SA-β-gal–positive cells were observed. However, in the YRS +100 ng/mL Wnt 3a group, the number of SA-β-gal–positive cells obviously increased. Scale bar = 25 µm. (B) Quantification of SA-β-gal–positive cells. The number of SA-β-gal–positive cells was not significantly increase in the YRS +10 ng/mL Wnt 3a and the YRS +50 ng/mL Wnt 3a groups compared with that in the YRS group (22.4±5.5 or 24.6±8.7 vs. 20.8±4.2. P > 0.1). However in the YRS +100 ng/mL Wnt 3a group, the number of SA-β-gal–positive cells (56.2±12.1) was significantly increased compared with that in the YRS group (*P<0.01). n = 5. (TIF) [file pone.0021397.s002.tif]

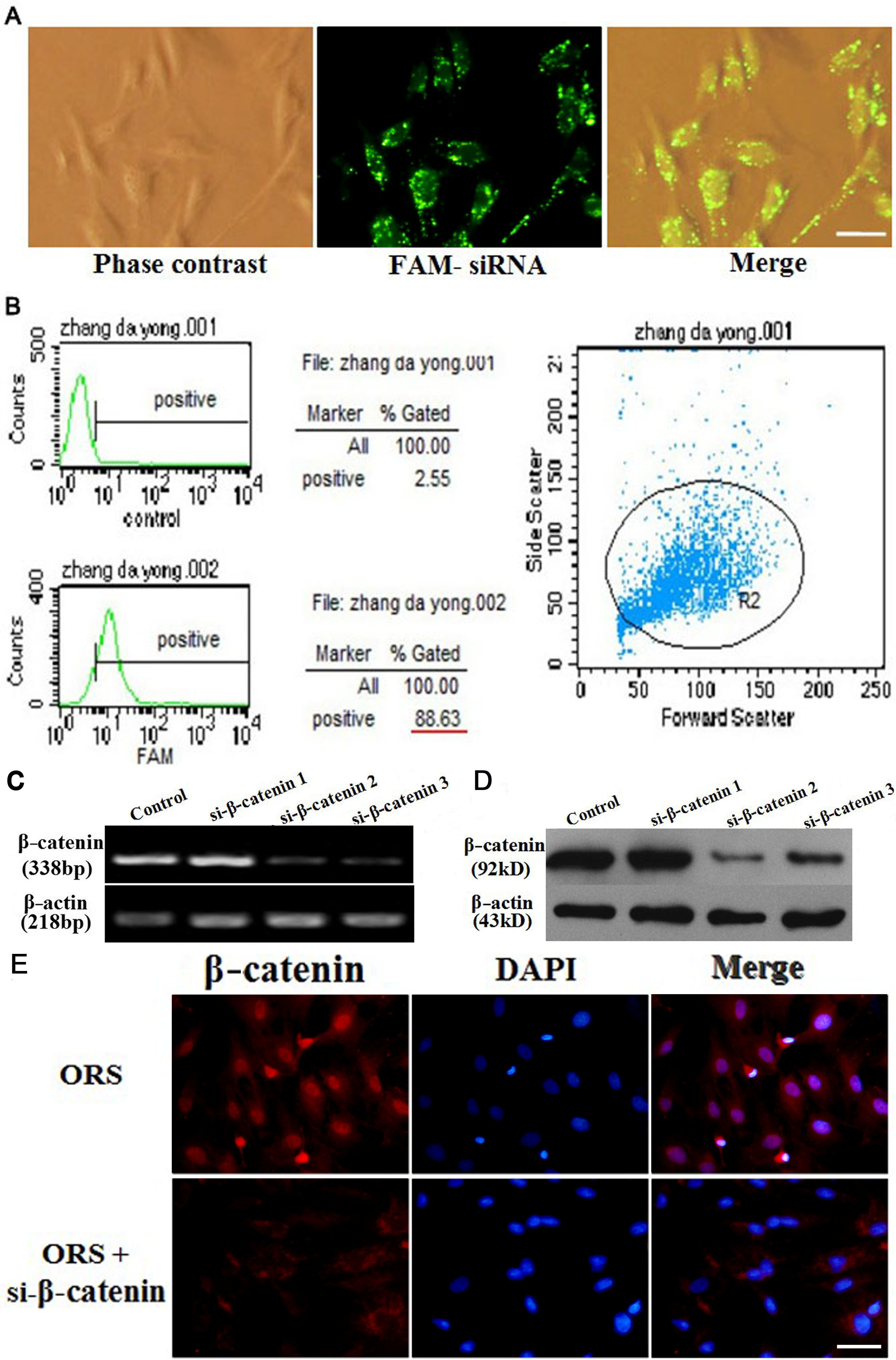

Supplement: Figure S3 — Detection of si-β-catenin transfection efficiency. (A) Up to 500 ng of FAM-labeled NC-siRNA was transfected into the MSCs for 12 h. Transfection efficiency was detected through phase-contrast and fluorescence microscopy. The results showed that siRNA had entered the cells. Scale bar = 25 µm. (B) Flow cytometry was also employed to detect the transfection efficiency of siRNA. The results were analyzed with Win MDI29 software, which indicated that siRNA transfection efficiency in MSCs was 88.63%. (C) RT-PCR analysis of β-catenin expression for screening the effective siRNA fragment. Three siRNA oligonucleotide targets were designed and synthesized to silence β-catenin (si-β-catenin1: 5′-GCTGACCAAACTGCTAAAT-3′; si-β-catenin2: 5′-CACCTCCCAAGTCCTTTA T-3′; si-β-catenin3: 5′-GCACCATGCAGAATACAAA-3′). MSCs were incubated with the control siRNA and three si-β-catenin for 12 h. The MSCs were further exposed to ORS for 12 h. β-catenin mRNA expression was examined by RT-PCR. The results show that β-catenin mRNA expression is significantly inhibited by si-β-catenin2 and si-β-catenin3. β-Actin was used as the internal control. The methods for RNA extraction and RT-PCR were the same as described in the Materials and Methods section. β-CateninFwd (5′-ACAGCACCTTCAGCACTCT-3′) and β-cateninRev (5′-AAGTTCTTGGCTATTACGACA-3′). (D) Western blot analysis of β-catenin expression for screening the effective siRNA fragment. After transfection with si-β-catenin for 48 h, the total β-catenin levels were assessed through western blot analysis. β-Actinoligonucleotide was used as the internal control. For the whole-cell extracts, the expression of β-catenin protein is significantly inhibited by si-β-catenin2 and si-β-catenin3. The si-β-catenin2 had a more efficient silencing effect. Thus, the si-β-catenin used in the present study was si-β-catenin2. (E) Immunofluorescence staining of β-catenin. After transfected with si-β-catenin for 48 h, MSCs were fixed with formaldehyde, stained for β-catenin (r [file pone.0021397.s003.tif]

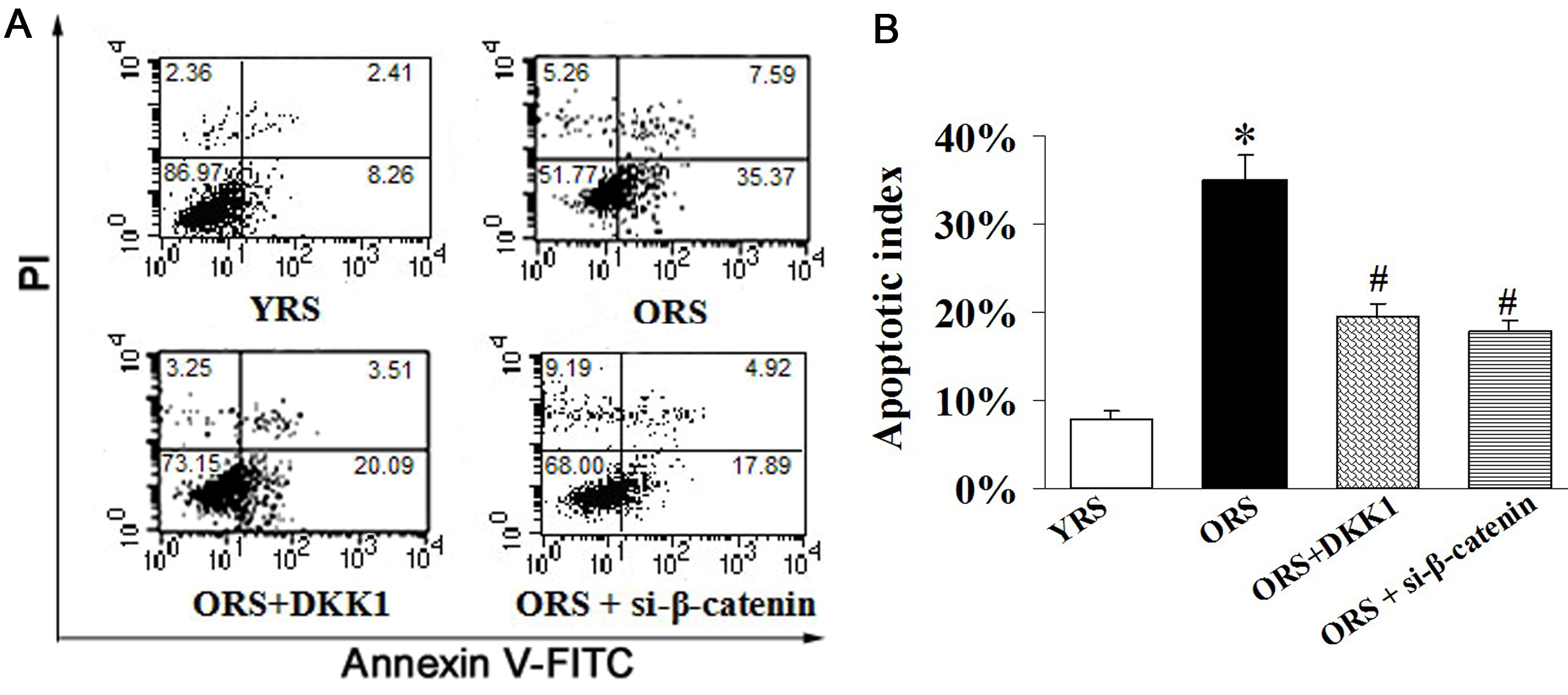

Supplement: Figure S4 — Flow cytometry analysis of apoptotic cells stained with Annexin V and PI. (A) Representative graphs of flow cytometry analysis. To further examine the apoptosis, the apoptotic cells were determined using an Annexin V/PI apoptosis detection kit (Sigma, USA) for flow cytometry (Calibur, BD Biosciences, USA), according to the manufacturer's instructions. Up to 2 × 10^4^ cells for each sample were analyzed using CellQuest software. Every experiment was performed in quadruplicate. (B) The apoptotic index of different groups according to annexin V/PI staining. The apoptotic index clearly increased in the ORS group compared with that in the YRS group (*P<0.01). However, after treatment with DKK1 or si-β-catenin in ORS to inhibit Wnt/β-catenin signaling, the apoptotic index significantly decreased (# P<0.01). n = 4. (TIF) [file pone.0021397.s004.tif]
